# Structure and Function Relationship of Activated Retinal Glia in Primary Open-Angle Glaucoma Patients

**DOI:** 10.1155/2017/7043752

**Published:** 2017-06-27

**Authors:** Christoph Nützi, Andreas Schötzau, Matthias C. Grieshaber

**Affiliations:** ^1^Department of Ophthalmology, Glaucoma Service, University of Basel, Basel, Switzerland; ^2^Center for Biomedical Statistics, University of Basel, Basel, Switzerland

## Abstract

**Purpose:**

To evaluate clinically activated retinal astrocytes and Müller cells (ARAM) regarding retinal sensitivity and retinal nerve fiber layer (RNFL) thickness in primary open-angle glaucoma (POAG).

**Methods:**

Central visual field (VF; i.e., retinal sensitivity) was measured with a custom-made macular pattern by microperimetry and correlated with the presence (ARAM+) or absence (ARAM−) of ARAM on red-free fundus photography and with the corresponding RNFL by optical coherence tomography (OCT).

**Results:**

In the eyes of POAG patients, ARAM+ had overall a significantly lower retinal sensitivity (ARAM+: 7.34 dB, ARAM−: 11.9 dB; *p* < 0.001) and lower RNFL thickness in the inferior peripapillary quadrants compared to ARAM− (RNFL superior: ARAM+ 74.2 μm, ARAM− 77.5 μm; RNFL temporal: ARAM+ 46.8 μm, ARAM− 53.0 μm, *p* < 0.001; and RNFL inferior: ARAM+ 63.2 μm, ARAM− 73.1 μm, *p* < 0.001). Within the same eye, ARAM+ showed a lower retinal sensitivity compared to ARAM− ([ARAM− (11.13 dB)] − [ARAM+ (9.56 dB) = 1.57 dB; *p* = 0.25). The proportion of ARAM+ per eye correlated strongly with reduced retinal light sensitivity (*p* = 0.02), corresponding lower peripapillary RNFL thickness (*p* = 0.02), and lower RNFL temporal quadrant thickness (*p* < 0.01), but not with greater age (*p* = 0.45).

**Conclusion:**

ARAM was more frequently identified in the eyes with a lower retinal sensitivity and peripapillary RNFL thickness and may be a clinical sign in the macula for an advanced stage of POAG.

## 1. Introduction

Glaucomatous optic neuropathy (GON) is characterized by typical changes of the optic nerve head (ONH) and corresponding visual field defects [1]. Elevated intraocular pressure (IOP) and low ocular perfusion pressure are the main risk factors for the development and progression of GON [2]. Morphologically, GON implies not only loss of retinal ganglion cells and their axons but also activation of glial cells and tissue remodeling. While the ophthalmic examination focuses on changes of the ONH like increased cup-disc ratio, peripapillary atrophy, and disc hemorrhage [3], morphological alterations in the retina due to activation of the glia were described in primary open-angle glaucoma (POAG) patients earlier [4–6].

Astrocytes and Müller cells form the macroglia in the retina. They both have structural, metabolic, and other supporting functions for neurons; once activated due to ischemic or other neuronal injuries of the retina [7], they show alterations in the morphology and gene expression [8, 9]; a characteristic upregulation of glial fibrillary acid protein (GFAP) was demonstrated in the human glaucomatous retinas [10] by immunohistochemistry. The clinical correlation of activated retinal astrocytes and Müller cells (termed ARAM) in glaucomatous eyes was described earlier [4–6, 11]. ARAM can be best visualized for their increased light scattering with scanning laser ophthalmoscopy using a green argon laser [6]. In red-free light, ARAM appear as patchy, discrete glittering but transparent alterations of the retina. The distribution and size of ARAM vary and do not conform to the orientation of the fiber nerve bundles. As a further characteristic, ARAM do not cause visual disturbance like metamorphopsia or reduce visual acuity [4–6]. Although clinically well described, there is no knowledge about structure-function correlations of ARAM in POAG at present.

Microperimetry measures the retinal light sensitivity, while simultaneously viewing the fundus, for example, fundus-controlled or fundus-driven perimetry [12]. The built-in eye-tracking system allows for automatic compensation of fixation movements. At the end of the visual assessment, a color retinography is superimposed on the retinal sensitivity map that enables an objective and precise comparison of retinal morphological changes with retinal sensitivity at any defined point [12].

The goal of this study was to quantify the distribution of ARAM and to correlate them with retinal light sensitivity and the corresponding peripapillary retinal nerve fiber layer (RNFL) thickness by optical coherence tomography (OCT).

## 2. Patients and Methods

Glaucoma patients were prospectively enrolled from the Glaucoma Service, Department of Ophthalmology, University Hospital of Basel, Switzerland. The study was accepted by the local ethics committee and followed the tenets of the Declaration of Helsinki.

### 2.1. Study Population

The study included POAG patients with ARAM, defined as patchy, discrete glittering but transparent alteration of the retina [4–6]. These morphological changes of the retina were identified and documented with red-free fundus photography (Carl Zeiss, Jena, Germany) by a masked operator after dilating the iris with phenylephrine and tropicamide.

Patients with a secondary cause of glaucomatous optical neuropathy such as steroids, pseudoexfoliation, and pigmentary dispersion; patients with a history of chronic or recurrent secure inflammatory eye disease like scleritis or uveitis; patients with a history of ocular trauma or intraocular surgery; and patients with clinical evidence for other retinal diseases such as age-related degeneration or diabetic retinopathy were excluded from the study.

Each POAG patient with ARAM that qualified for the study had a complete ophthalmologic examination. Patients with untreated IOP equal or less than 21 were classified as normal-tension glaucoma (NTG), and others as high-tension glaucoma (HTG). Further, the patients were characterized as primary vascular dysregulation (PVD) [13], if they were clearly familiar with the history of frequent cold hands, even during summer time.

### 2.2. Microperimetry

**Figure 1:**
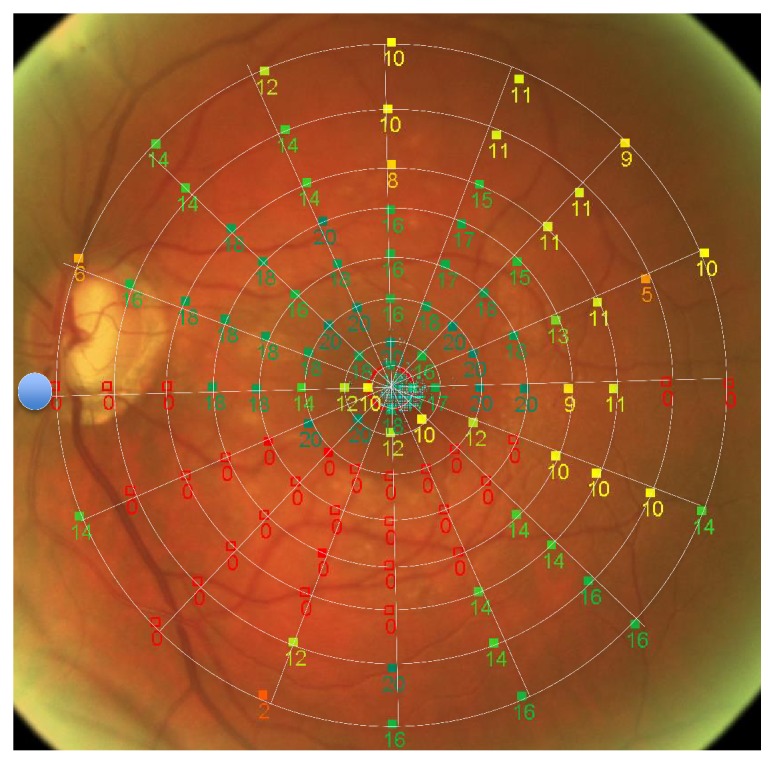
Custom-made macular visual field (VF) pattern with 108 testing points by microperimetry testing retinal sensitivity (example of a left eye).

**Figure 2:**
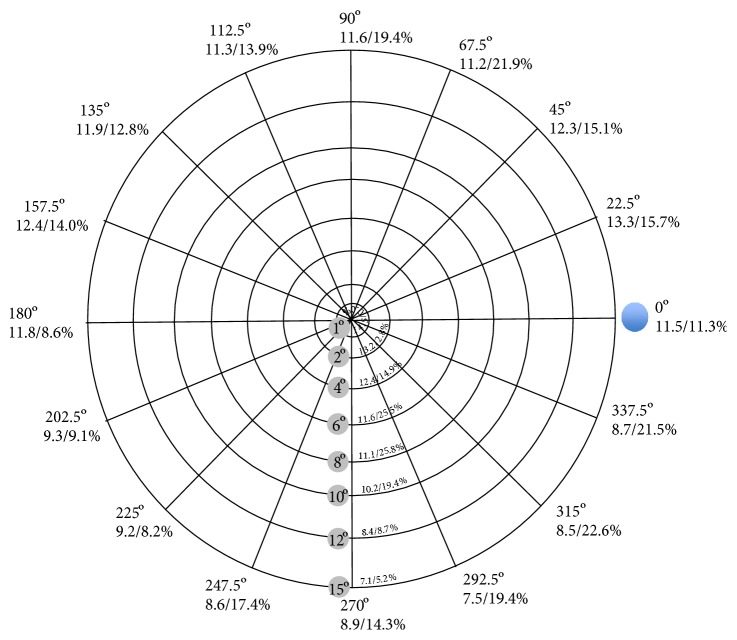
Visual field (VF) pattern with 1° to 15° circle around the fovea and 16 lines spread out from the center of the fovea every 22.5 degrees with their mean values of microperimetry (dB) and ARAM+ (in percentage (%), based on ARAM+ and ARAM−): microperimetry/ARAM+ in %.

**Figure 3:**
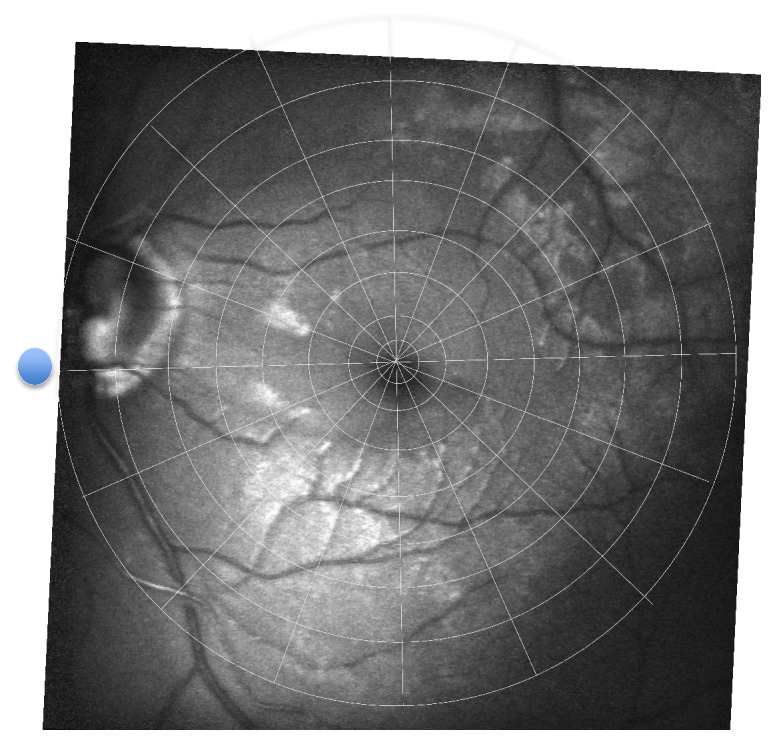
Red-free fundus photography with superimposed visual field pattern showing the presence (ARAM+) or absence (ARAM−) of ARAM at each testing point.

Automated microperimetry (NIDEK MP-1, Nidek Technologies, Padua, Italy) was used for testing the retinal sensitivity (from 0 dB up to maximum 20 dB) with a Goldmann III stimulus (200 ms) and a 4–2 dB threshold strategy. The background luminance was 1.27 cd/m^2^ (=4 asb). We created a specific VF pattern to represent the macular region with 108 testing points located on eight circles (1°, 2°, 4°, 6°, 8°, 10°, 12°, and 15°) around the center of the fovea, where 16 lines spread out every 22.5 degrees (Figures 1() and 2()). The innermost circle consisted of 4 points, the second circle of 8 points, and the remaining circles each of 16 points; thus, every single testing point was readily identifiable as a coordinate by the degree of eccentricity (distance to fovea) and by the degree of the drawn line originating from the fovea center (defined as 0° from fovea to optic disk, 90° from fovea to superior, 180° from fovea to temporal, and 270° from fovea to inferior) (Figure 2()). The testing points, arranged in a fixed VF pattern in the retinal sensitivity map, were compared to the localization of ARAM in the following way; first, we superimposed the red-free fundus photography on the colored image of the retina automatically generated by the microperimetry. Thereafter, we grouped the VF pattern and the red-free fundus photography as a new compound image. This allowed us to localize ARAM precisely according to the coordinates of the VF pattern. The VF pattern was side mirrored to take the eye side (right and left) into account. In addition, the presence or absence of ARAM was evaluated for each testing point of the VF pattern (*n* = 108). Test locations with ARAM were defined as ARAM+, and test locations without ARAM as ARAM− (Figure 3()). For quality reasons, testing points which were too dark or were out of the image section were not analyzed.

### 2.3. OCT Imaging

**Figure 4:**
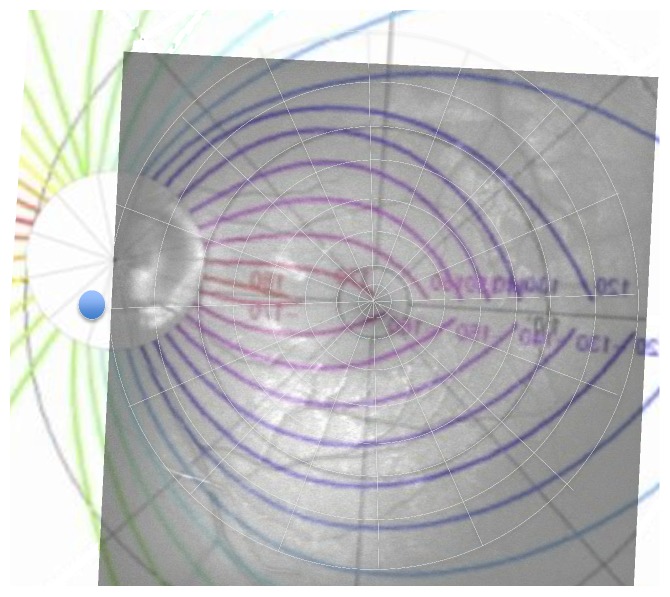
Nerve fiber course model (Jansonius et al.) with superimposed visual field (VF) program to analyze the corresponding RNFL sector OCT value referring to the VF patterns points.

Fast RNFL thickness OCT scan (Stratus OCT, Version 4.0.4, Zeiss, Oberkochen, Germany) was used to quantify peripapillary RNFL thickness. This scan type acquired three peripapillary scans, each consisting of 256 A-scans along a diameter of 3.4 mm around the center of the optic disc. The average of the RNFL thickness for the twelve clock-hour sectors was automatically calculated. Jansonius et al. [14] developed a mathematical model describing the physiological course of retinal fiber bundle trajectories in the human eyes. We superimposed the fiber bundle course model on red-free fundus photography by matching the fovea, the 10° circle surrounding the fovea, and the center of optic disc to make sure that the sectors of the OCT scan refer to the corresponding points of the VF pattern on the red-free fundus photography (Figure 4). We draw a proportional 3.4 mm diameter centered in the optic disc. Then, each point of the VF pattern of the red-free fundus image referred exactly to one of the twelve peripapillary sectors according to the course of the retina fiber bundle, which in turn allowed to correlate with ARAM on the corresponding peripapillary RNFL sector thickness.

### 2.4. Statistical Methods

Descriptive statistics (mean, standard deviation (SD), and percentage) were used to determine continuous variables (demographic data, ARAM+ and retinal sensitivity in dB, and RNFL in microns). Comparisons within descriptive statistics were made using *t*-tests or Fisher's exact tests. Linear mixed effects models were performed to compare the RNFL thickness and retinal sensitivity (microperimetry) between ARAM+ and ARAM− within each eye. This kind of models is a suitable tool to analyze repeated measure data. The results are presented as differences of means with the corresponding *p* values. Correlations between OCT sectors were estimated using the Spearman correlation. A *p* value < 0.05 was considered significant. All calculations were done using the statistical software R project, version 3.1.1. [15].

## 3. Results

Out of 35 eyes, 18 eyes (10 right, 8 left) of 12 POAG patients (9 females, 3 males) with ARAM were included in this single-center study (demographic data, Table 1). Not included were eyes from patients who were unable to reliably perform microperimetry examination due to poor vision (*n* = 4), eyes with missing OCT data (*n* = 3), eyes with poor quality of the red-free fundus photography (*n* = 2), and eyes where red-free fundus photography showed too punctual changes (*n* = 3) or changes without sharp limits (*n* = 5), which were not compatible with the abovementioned definition of ARAM in a second look.

The proportion of ARAM+ was determined by ARAM+ in percentage (%) based on ARAM+ and ARAM− (ARAM+/(ARAM+ and ARAM−)) per eye or per circle, respectively. The proportion of ARAM+ increased continuously from 1° eccentricity to 6° and 8° from where it continuously decreased to 15° (*p* < 0.01) (Figure 2, Table 2). The distribution of ARAM+ proportion along the 16 lines spread out from the fovea ranging between 8.2% and 22.6% (*p* = 0.03). The mean retinal sensitivity decreased continuously from the center of 1° circle to the outmost 15° circle (*p* < 0.01). ARAM+ had overall a significant lower retinal sensitivity compared with ARAM− (ARAM+: 7.34 dB, ARAM−: 11.9 dB; *p* < 0.001) (Table 3). The proportion of ARAM+ per eye correlated inversely with retinal sensitivity—the higher the proportion of ARAM+, the lower the retinal sensitivity (*p* = 0.02). The pairwise difference of mean retinal sensitivity between ARAM+ and ARAM− within the same eye was not significant ([ARAM− (11.13 dB)] − [ARAM+ (9.56 dB)] = 1.57 dB; *p* = 0.25). However, when the test location of ARAM of a given circle around the fovea (1° to 15° circle) was taken into account, ARAM+ showed a significant lower retinal sensitivity than ARAM− (*p* = 0.0017) (Figure 2, Table 3).

Overall, ARAM+ had a corresponding significant lower thickness in the superior (*p* = 0.02), temporal (*p* < 0.001), and inferior (*p* < 0.001) peripapillary RNFL quadrants compared with ARAM− (Table 4). Likewise, the proportion of ARAM+ per eye correlated inversely and significantly with the mean corresponding RNFL sector thickness (*p* = 0.02); the higher the proportion of ARAM+ per eye was, the smaller the mean corresponding RNFL sector thickness. In addition, ARAM+ proportion per eye correlated strongly with the temporal quadrant (*p* < 0.01) and slightly with the inferior quadrant (*p* = 0.04), but not with the superior (*p* = 0.51), and nasal quadrant (*p* = 0.75).

## 4. Discussion

In this study, test locations of ARAM+ had overall a significant lower retinal sensitivity and corresponding lower thickness in the superior, temporal, and inferior peripapillary RNFL quadrants, compared to test locations of ARAM−. Of note, ARAM+ showed a lower retinal sensitivity compared to ARAM− even within the same eye. Furthermore, the proportion of ARAM+ per eye correlated strongly with both reduced retinal light sensitivity and corresponding peripapillary RNFL thickness, in particular the temporal quadrant.

The distribution of ARAM in the retina pursuant to the VF pattern had a large range. The innermost circle had no ARAM and represents well the foveal avascular zone, which has no astrocytes and only extremely elongated outer trunks of Müller cells. With the exception of the avascular zone, both (nonactivated) glial cells are distributed consistently throughout the entire retina and decline toward the periphery [16, 17]. In this study however, we determined in which areas of the macula glial cells were predominantly activated and quantified them. Further and in line with previous studies [4, 5], we measured the highest proportion of ARAM+ 6 and 8 degrees away from the center describing the perifoveal zone. From the perifoveal zone, the proportion of ARAM decreased continuously to the center and to the outmost circle referring 15 degrees away from the fovea center.

The proportional distribution of ARAM+ along the circles was very different from the distribution of retinal light sensitivity in all testing points. Overall, retinal sensitivity decreased continuously from the innermost to the outmost position of the VF pattern according to the sensitivity relative to the normal hill of vision. However, ARAM+ had overall a significant lower retinal sensitivity, compared with ARAM− (ARAM+: 7.34 dB, ARAM−: 11.9 dB; *p* < 0.001).

Within the same eye, there was a trend toward lower retinal sensitivity in ARAM+ compared to that in ARAM−. More differentiated and adjusted for the circle, ARAM+ showed significant lower retinal sensitivity of the same circle around the fovea compared to ARAM− (*p* = 0.0017). Therefore, the distance to fovea can be excluded as a confounding factor for a lower retinal sensitivity of ARAM+. A normative MP-1 value study with healthy subjects identified age as an influencing factor for the mean light sensitivity threshold. The oldest age group (70–75 years), which is comparable with our study group (77.6 years), showed a mean light sensitivity threshold of 18.6 dB [18].

Further, we investigated whether lower retinal sensitivity is an overall effect of a larger amount of ARAM+ in a given eye and found that the proportion of ARAM+ per eye correlated well and inversely with the retinal sensitivity; the higher the proportion, the more decreased the retinal sensitivity was on average. Thus, we suggest that a high density of ARAM+ in an eye may be a clinical and structural sign for a low retinal sensitivity in eyes with POAG.

Besides functional relationship, we studied the association of ARAM with the structure determined by the RNFL. As previously found, the variability of the RNFL thickness in the macula region by OCT may be masked by the presence of the ARAM areas [19]. Therefore, we used the *peripapillary* RNFL OCT scan and correlated the VF testing points with the corresponding RNFL sector according to the course of the nerve fiber bundle. This method allowed us to evaluate the influence of ARAM on the corresponding peripapillary RNFL thickness more objectively. The corresponding mean peripapillary RNFL sector per eye was 63.1 *μ*m. Overall, ARAM+ had significant lower superior, temporal, and inferior mean RNFL sector thickness compared with ARAM−. Additionally, the proportion of ARAM+ per eye correlated significantly with the corresponding mean RNFL sector thickness; the higher the proportion of ARAM+ was, the smaller the corresponding mean RNFL sector thickness. In this way, a higher proportion of ARAM+ can be interpreted as a clinical and structural sign for a lower corresponding peripapillary RNFL thickness and, in other words, for a more advanced stage of glaucoma.

The significance of the correlation between ARAM+ and lower peripapillary RNFL thickness is underlined by the fact that the highest distribution of ARAM and VF testing points referred to the temporal quadrant. In this quadrant, the high proportion of ARAM+ per eye correlated strongly with the thickness of RNFL in contrast to the superior and inferior quadrant with fewer ARAM and VF testing points. Thus, the more nerve fiber bundles were assigned to ARAM+, the higher the effect on the peripapillary RNFL quadrant was, suggesting that ARAM may have some local interaction with nerve fibers resulting in lower corresponding RNFL thickness. Importantly, the proportion of ARAM+ per eye did not correlate with greater age (*p* = 0.45) that confirms findings of a previous study [6]. Therefore, ARAM cannot be classified as an age-related effect.

As somehow expected, the VF function correlated well with the RNFL structure. The higher the mean retinal sensitivity of a given VF pattern was, the thicker the corresponding mean RNFL sector (*p* < 0.01). This underlines the reliability of the analyses.

Both astrocytes and Müller cells surround blood vessels at the surface of the retina with their end-foot processes showing their close connection to retinal vasculature [20, 21]. Previous studies found a significant influence of PVD on the presence of ARAM in POAG patients [6] explaining the high amount of PVD patients (75%) in our study group, when only POAG patients with ARAM were included. PVD is more frequent in female patients [13], representing the bigger amount of females in our study group. Patients with PVD are predisposed to respond inadequately to various stimuli such as coldness, emotional stress and others [13]. The disturbed autoregulation leads to instable ocular blood flow and via repeated mild reperfusions injury to oxidative stress which contributes to the pathogenesis of glaucomatous optic neuropathy [22]. Considering that IOP fluctuation at a high level especially in combination of disturbed autoregulation is related to unstable blood flow [23], a link can be made between elevated pressure and ARAM in POAG eyes.

In this study, there were some drawbacks. First, the peripapillary OCT scan measures the RNFL thickness remotely from the location of ARAM, and thus, no true comparison between the RNFL of ARAM+ and ARAM− can be made. The peripapillary RNFL measurement can be considered as a surrogate or approximation, as only the portion of ARAM+ having an impact on the corresponding RNFL sector according to the course of retinal fiber bundle was analyzed. Although ARAM may compensate or mask RNFL loss in a thickness measurement scan like OCT of the macula region [19], it has to be proved in further studies, whether new high-resolution OCT may be useful to differentiate between RNFL and ARAM in the macula region. Second, although all patients had experience in VF exams, microperimetry was performed only once. Thus, fluctuations, test-retest variability, and so on, having an impact on the retinal sensitivity, were not assessed. Third, even though the VF pattern of the microperimetry (*n* = 108) analyzed a big amount of VF testing points per eye, the study population of 18 eyes was rather small.

In conclusion, we identified a large quantity of ARAM+ in POAG as a clinical sign for an advanced stage of glaucoma associated with a lower retinal sensitivity and lower corresponding peripapillary RNFL. ARAM may represent a local and limited response to reduced RNFL thickness and retinal sensitivity. Further studies are needed to assess whether ARAM interacts with RNFL in a neuroprotective or in a neurodegenerative way and to evaluate the impact of oxidative stress on the activation of macroglial cells in glaucomatous eyes.

## Figures and Tables

**Table 1:** Descriptive and demographic data of the cohort.

*Patients (n* = 12)	
Age (in years)	77.6 (7.2)
Sex (w; m)	9 (75%); 3 (25%)
PVD (yes; no)	9 (75%); 3 (25%)
*Eyes (n* = 18)	
Side (left; right)	8 (44.4%); 10 (55.6%)
Disease (NTG; HTG)	4 (22.2%); 14 (77.8%)
*RNFL quadrants*	
RNFL superior	76.7 *μ*m (22.8)
RNFL temporal	51.6 *μ*m (14.5)
RNFL inferior	70.8 *μ*m (28.3)
RNFL nasal	55.9 *μ*m (14.1)
*Pattern points*	
ARAM (+; −)	269 (15.5%); 1468 (84.5%)
Microperimetry (*n* = 1944)	10.5 dB (7.8)
Corresponding RNFL sector (*n* = 1862)	63.1 *μ*m (28.2)

**Table 2:** Descriptive data of visual field pattern: ARAM+, ARAM−, and mean microperimetry value.

Visual field pattern	ARAM		Microperimetry (dB) (SD)
	ARAM+ (%)	ARAM−	
Overall (*n* = 1944)	269 (15.5%)	1468	10.5 (7.8)

*Distance from fovea in degree* (°)			
1 (*n* = 72)	0 (0.0%)	71	14.5 (5.7)
2 (*n* = 144)	4 (2.8%)	137	13.2 (7.2)
4 (*n* = 288)	42 (14.9%)	240	12.4 (8.2)
6 (*n* = 288)	71 (25.5%)	207	11.6 (8.1)
8 (*n* = 288)	70 (25.8%)	201	11.1 (7.9)
10 (*n* = 288)	51 (19.4%)	212	10.2 (7.5)
12 (*n* = 288)	21 (8.7%)	218	8.4 (7.3)
15 (*n* = 288)	10 (5.2%)	182	7.16 (6.8)
*n*		1737	1944
*p* overall		<0.001	<0.001

*Lines from fovea in degree* (°)			
0 (*n* = 144)	13 (11.3%)	102	11.5 (7.8)
22.5 (*n* = 180)	16 (15.7%)	86	13.3 (6.4)
45 (*n* = 126)	19 (15.1%)	107	12.3 (6.8)
67.5 (*n* = 180)	23 (21.9%)	82	11.2 (7.0)
90 (*n* = 144)	25 (19.4%)	104	11.6 (6.6)
112.5 (*n* = 180)	14 (13.9%)	87	11.3 (6.7)
135 (*n* = 126)	15 (12.8%)	102	11.9 (6.7)
157.5 (*n* = 180)	13 (14.0%)	80	12.4 (6.7)
180 (*n* = 144)	10 (8.6%)	106	11.8 (8.1)
202.5 (*n* = 180)	7 (9.1%)	70	9.3 (8.4)
225 (*n* = 126)	8 (8.2%)	90	9.2 (8.6)
247.5 (*n* = 180)	15 (17.4%)	71	8.6 (8.3)
270 (*n* = 144)	19 (14.3%)	114	8.9 (8.4)
292.5 (*n* = 180)	21 (19.4%)	87	7.5 (8.4)
315 (*n* = 126)	28 (22.6%)	96	8.5 (8.6)
337.5 (*n* = 180)	23 (21.5%)	84	8.7 (8.2)
*n*		1737	1944
*p* overall		0.026	<0.001

**Table 3:** Comparing microperimetry values of ARAM+ with those of ARAM− from the same distance to fovea (1° to 15° circle) within the eye.

Circle°	Lower 95%	Estimate	Upper 95%	*p* value
1	NA	NA	NA	NA
2	−1.98	3.99	9.95	0.189
4	−5.80	−2.69	0.42	0.089
6	−5.24	−2.54	0.15	0.0636
8	−5.67	−2.98	−3.0	0.297
10	−4.73	−1.93	0.86	0.174
12	−4.37	−1.27	1.84	0.421
15	−4.17	−0.16	3.85	0.937

NA: not applicable (no ARAM+ at circle 1°).

**Table 4:** Microperimetry and peripapillary OCT RNFL of superior, inferior, temporal, and nasal quadrants of ARAM+ were compared with those of ARAM−.

ARAM+ and ARAM−	ARAM+	ARAM−	*p* overall
*n* = 1737	*n* = 269	*n* = 1468	
Microperimetry (dB) (SD)	7.3 (7.7)	11.9 (7.5)	<0.001
RNFL superior (*μ*m) (SD)	74.2 (21.1)	77.5 (23.1)	0.021
RNFL temporal	46.8 (12.7)	53.0 (14.9)	<0.001
RNFL inferior	63.2 (22.6)	73.1 (30.0)	<0.001
RNFL nasal	55.3 (13.5)	55.9 (14.7)	0.515
